# Disruption of Dopamine Homeostasis Associated with Alteration of Proteins in Synaptic Vesicles: A Putative Central Mechanism of Parkinson’s Disease Pathogenesis

**DOI:** 10.14336/AD.2023.0821-2

**Published:** 2024-05-07

**Authors:** Xuanxiang Jin, Xiaoli Si, Xiaoguang Lei, Huifang Liu, Anwen Shao, Lingfei Li

**Affiliations:** ^1^The First School of Medicine, School of Information and Engineering, Wenzhou Medical University, Wenzhou, Zhejiang, China.; ^2^Department of Neurology, the Second Affiliated Hospital, School of Medicine, Zhejiang University, Hangzhou, China.; ^3^Department of Neurology, First Affiliated Hospital of Kunming Medical University, the First School of Clinical Medicine, Kunming Medical University, Kunming, China.; ^4^Division of Neurology, Department of Medicine, University of Hong Kong, Hong Kong.; ^5^Department of Neurosurgery, The Second Affiliated Hospital, School of Medicine, Zhejiang University, Hangzhou, China.; ^6^Key Laboratory of Precise Treatment and Clinical Translational Research of Neurological Disease, Hangzhou, China.; ^7^Department of Neurology, Affiliated Hangzhou First People’s Hospital, Zhejiang University School of Medicine, Hangzhou, China.

**Keywords:** Parkinson’ disease;, genetics, synaptic vesicle proteins, presynaptic dopamine homeostasis

## Abstract

Vestigial dopaminergic cells in PD have selectivity for a sub-class of hypersensitive neurons with the nigrostriatal dopamine (DA) tract. DA is modulated in pre-synaptic nerve terminals to remain stable. To be specific, proteins at DA release sites that have a function of synthesizing and packing DA in cytoplasm, modulating release and reingestion, and changing excitability of neurons, display regional discrepancies that uncover relevancy of the observed sensitivity to neurodegenerative changes. Although the reasons of a majority of PD cases are still indistinct, heredity and environment are known to us to make significant influences. For decades, genetic analysis of PD patients with heredity in family have promoted our comprehension of pathogenesis to a great extent, which reveals correlative mechanisms including oxidative stress, abnormal protein homeostasis and mitochondrial dysfunction. In this review, we review the constitution of presynaptic vesicle related to DA homeostasis and describe the genetic and environmental evidence of presynaptic dysfunction that increase risky possibility of PD concerning intracellular vesicle transmission and their functional outcomes. We summarize alterations in synaptic vesicular proteins with great involvement in the reasons of some DA neurons highly vulnerable to neurodegenerative changes. We generalize different potential targets and therapeutic strategies for different pathogenic mechanisms, providing a reference for further studies of PD treatment in the future. But it remains to be further researched on this recently discovered and converging mechanism of vesicular dynamics and PD, which will provide a more profound comprehension and put up with new therapeutic tactics for PD patients.

## 1. Introduction

### 1.1 Background on PD

PD is the second neurodegenerative disease in incidence [1]. Its pathological process is featured with a progressive loss of midbrain dopaminergic neurons, ultimately resulting in severe striatal DA shortage and primary movement disorders, such as resting, tremor and bradykinesia [1]. However, the potential molecular mechanism of the loss of dopaminergic neuron remains doubtful and the curable treatment to stop disease progression is under the radar.

### 1.2 Significance of dopamine homeostasis in PD pathogenesis

It is reported that midbrain dopaminergic neurons are responsible for DA release, mainly locating in the substantia nigral (SN) and the ventral tegmental area (VTA) [2]. DA is a highly active antioxidant and accumulation of cytosolic DA will induce oxidative stress-related damage by the generation of reactive species, serving as a basis for the high vulnerability to genetic and environmental damage [3, 4]. Clinical studies have found Abnormalities in DA turnover in early-stage PD patients [5] and 87-90% reduction of DA storage in idiopathic PD patients [6]. Disruption of DA homeostasis triggers a series of important cell signalling pathways related to PD in the downstream, such as mitochondrial and lysosomal dysfunction [7], α-synuclein polymerization [8], and eventually results in the death of dopaminergic neurons [9].

### 1.3 synaptic vesicles (SVs) and their role in dopamine regulation

SVs were first found in nerve terminals along with the development of electron microscope in the early 1950s [10]. SVs contain different neurotransmitters and are held in axon terminals. When the voltage-dependent calcium channel open, neurotransmitters are released to synaptic cleft to propagate nerve signals. Initially, SVs were considered simple in structure because only a few proteins met the condition of a sphere of 40 nm diameter until Takamori pointed out that SVs contained approximately 40 types of transmembrane proteins [11-13]. SVs contain two kinds of transport proteins involved in neuro-transmitter uptake and SV exocytosis, endocytosis, and recycling. Up to now, a variety of structural and functional changes in nerve terminals, such as depletion of SV and synaptic proteins, Ca^2+^ dynamics impairment, have been found in neurological diseases and thought to be the very early changes of neurological diseases [14]. Probably it accounts for an initial pathophysiological event which disrupts DA homeostasis in presynaptic nerve terminals and finally contributes to the death of dopaminergic neurons. Some of the familial factors of PD such as α-synuclein, leucine-rich repeat kinase 2 (LRRK2), parkin, PTEN-induced kinase 1 (PINK1), and DJ-1, serve to modulate synaptic function and their missense mutants triggers synaptic dysfunctions [15-19].

In summary, dopaminergic synaptic vesicles (DA-SVs) are responsible for storage and transportation of DA in DA neurons. Abnormalities in these DA-SVs result in imbalanced DA homeostasis, which trigger a series of pathological processes and finally cause the death of DA neurons. In this review, we summarize abnormalities found in the composition and function of SVs from both PD patients and basic research models. We also review the connection between deficiency in SVs and the current molecular mechanisms of PD pathogenesis.

## 2. Disruption of DA Homeostasis in Parkinson's Disease

### 2.1 Overview of DA dysregulation in PD

DA possesses a high reactivity and is usually stockpiled in SVs. Dissociative DA in the cytoplasm can oxidize itself to produce reactive oxygen species and toxic quinones [20]. There exist three neurotoxic mechanisms of dopaminergic neurodegenerative changes in PD-related SN pars compacta dopaminergic neurons triggered by DA oxidative products: 1) homeostatic imbalance of neuronal proteins in aspects of aggregation [21, 22], competition of functional modifications after translation (i.e. ubiquitination and acetylation) and accumulation of protein ubiquitination [23]; 2) enzyme repression [24, 25]; 3) indirect influences including oxidative stress , disorder of mitochondrial function [26]. Thus, DA homeostatic imbalance makes a significant impact in the multi-molecular mechanisms of the PD onset.

### 2.2 Importance of SVs in maintaining DA homeostasis

The compositions and sites of SVs vary with their neuronal phenotype and cargo, mainly including Golgi-derived small SVs, large dense core vesicles, and tubule-vesicular structures resembling smooth ER stores. [27]. DA-SVs release DA from stomata and/or dendrites of DA neurons. Disruption of DA-SVs structure and composition affects sequestration of DA causing oxidative stress and finally leading to cell death [28]. For instance, vesicular monoamine transporter type-2 (VMAT2, which is mostly located in the tubulovesicles), the vacuolar-type adenosine triphosphatases (V-ATPase, which is important for intraluminal acidification of both autophagy-lysosomes and SVs), the soluble N-ethylmaleimide-sensitive factor (NSF) attachment protein receptor (SNARE) proteins, vesicle-associated membrane protein (VAMP) and Syntaxin, the function of these DA-SV specific characterized proteins is of great significance for the fusion and release of SVs. Of note, the latest research has shown that in the random transformation of SV among three ATP-mediated ultralong-lived forms: proton-pumping, inactive and proton-leaky, V-ATPase can activate the loading of neurotransmitters and all-or-none random fluctuations of electrochemical gradient in SVs [2, 29]. Despite the proven dynamic and transformable molecular compositions of presynaptic and postsynaptic nerve endings, secular stability of the reaction is still indispensable to ensure long-lasting synaptic development and plasticity [30].

SV recycling includes several steps. First, SVs which are full of neurotransmitters dock to the release site, namely active zone. Then SVs prepare and fuse with the plasma membrane (exocytosis), followed by the sorting and retrieval of molecules inside vesicles (endocytosis) [31]. The pathway of SV recycling plays a decisive role in the modulation of neurotransmitter content and characteristics of release, such as the specialized machinery for DA secretion in striatum [32]. In addition, the organized release sites are probably not necessary which differ from fast synaptic trafficking, because neurotransmitter release and receptor activation mainly occur in active regions. Optogenetic experiments also revealed that DA release was fast and positive. It demonstrated that protein scaffolds existed, which promoted the inflow of coupling Ca^2+^ to vesicle fusion. Thus, sparse and mechanistically specialized active zone-like release sites are responsible for DA secretion, which provides support of precise coding for DA in space-time and molecular machinery for regulation [32]. It was also reported that the copy numbers of proteins in the same step of SV recycling had a close correlation [33]. By comparison, copy numbers ranged from about 150 copies of endosomal fusion proteins to more than 20,000 of the exocytotic ones, even surpassing three orders of magnitude between steps [33].

### 2.3 Evidence of altered proteins in SVs in PD

Protein alterations are commonly confirmed in PD. Among them, the changes of 3,4-dihydroxyphenyl-acetaldehyde (DOPAL) and α-synuclein have attracted much attention, which have been verified in many experiments. DOPAL was found to elevated in post-mortem brain of PD patients [34]. In the study of a PD model prepared by the injection of 12-hour 6-hydroxydopamine, DOPAL remarkably boomed in the striatum regions of rats, reaching about 120 ng/mg tissue [35]. Similar consequences also have been found in the rat model administered by rotenone, including damaged vesicle storage, inactive ALDH, and accumulative DOPAL. It revealed a decrease of 82% for DA and a raising of 1.69 times for DOPAL in the experimental group [36]. Moreover, a specific antibody was used to quantify the changes of α-synuclein affected by DOPAL, drawing a conclusion of the level of α-synuclein approximately in a range from 10 to 40 picogram per microgram of total proteins [37]. As for the connection between DOPAL and α-synuclein, to be specific, DOPAL inhibits the fibril formation of α-synuclein especially in the concentration of 20 μM and is in direct proportion to the aggregates of α-synuclein [38].

## 3. Role of Synaptic Vesicular Proteins in DA Homeostasis

### 3.1 Function and importance of synaptic vesicular proteins

DOPAL: One metabolic product of DA catalyzed by monoamine oxidase is DOPAL. The catechol and the aldehyde moieties of DOPAL are highly sensitive in collaboration to modify functional protein residues and aggregate proteins [21, 22, 39]. Its resulting effect on α-synuclein oligomerization is also proved to produce sodium dodecyl sulfate-resistant species in a high molecular weight which accounts for pathological relevance in PD [21, 23, 40]. Additionally, a recent survey has revealed that, in vitro, DOPAL serves to trigger modification of ubiquitin lysine and ubiquitin oligomerization. Especially in possession of seven functional lysine, ubiquitin can be polymerized to target proteins in various cellular compartments [21].

α-synuclein: α-synuclein is generated in the central nervous system as a presynaptic protein [41]. In a variety of structural forms including monomer [42], disordered nonamyloid oligomers, ordered amyloid oligomers [43], and fibrils [44, 45], it is not only the main ingredient of Lewy bodies, but also one of the characteristic histopathological performances of PD [30]. A structural set of the first 30 residues of α-synuclein binding to the surface of small unilamellar vesicles is consist of multiple acidic lipids including 1,2-dioleoyl-sn-glycero-3-phos-phoethanol-amine, 1,2-dioleoyl-sn-glycero-3-phospho-L-serine and 1,2-dioleoyl-sn-glycero-3-phosphocholine lipids in a 5:3:2 molar ratio [46]. In terms of α-synuclein itself, N-terminal acetylation modulates the affinity of combination between α-synuclein and SVs with no alteration in the structural features of the bound state. Meanwhile calcium combination in C-terminal also takes the effect of regulation [47, 48]. In physiological conditions α-synuclein can regulate SV proteins and membranes, control SVs transmission, assembly, and fission, alter lipid-selective and viscoelastic properties of negatively charged lipid vesicles, and trigger the inflammatory sensitization [44, 49-54]. In addition, Eguchi found that wild-type monomeric α-synuclein takes negative effects in vesicle endocytosis and synaptic fidelity by tubulin aggregation at the calyx of held [55]. In short, alterations of SV function vary with the mutation, expression level, and structure of α-synuclein. But up to now, how α-synuclein affects SV function has been still unrevealed. [56-59].

### 3.2 Abnormalities in synaptic vesicular protein expression and implications in DA release and reuptake

DOPAL: Proteins embellished by DOPAL do harm to enzyme activity. Possessing an accessible functional cysteine or lysine in an active site, the enzyme can be inactivated by DOPAL to trigger a significant result on the pathways of metabolism. For example, tyrosine hydroxylase (TH) is confirmed to be the target of DOPAL in a proteomic study in PC6-3 cells [60]. By TH purification and High Performance Liquid Chromatography quantification of Levodopa (L-DOPA), DOPAL is considered to modify lysine residues, resultantly triggering rearrangement of enzyme conformation and inactivity of 80-95% TH. Due to the limiting-velocity role of TH in the process of synthesizing DA from tyrosine, DA release is hindered by inactive TH in nigrostriatal circuits [61, 62]. Additionally, in a study concerning N27 dopaminergic cells, DOPAL was found to modify aldehyde moiety to restrain active glutathione S-transferase (GST), a frontline of resisting oxidative stress, which was unable to reverse and varied over time. A 24-hour incubation with 100 μM DOPAL even triggered entire inactivity of GST [63, 64].

Relevant studies show that DOPAL can be oxidated by H_2_O_2_ to generate hydroxyl radicals. Simultaneously its catechol group was prone to engender DA-analogous semiquinone radicals and ortho-quinones by autooxidation [65]. It is estimated that the resulting radical oxygen species (ROS) intensify neuronic oxidation damage, playing a negative impact on abnormal DNA structures, protein cross-linking and lipid overoxidation. In the study researched by Anderson, DOPAL was proved to be a substrate of Cyclooxygenase-2, which also sped up the oxidation of DOPAL catechol. On the contrary, superoxide dismutase decomposes superoxide anions into molecular oxygen and hydrogen peroxide to resist oxidation. It strengthens the cross-linking effect of DOPAL and associated protein to prevent lysine modification. Other antioxidants such as N-acetylcysteine, glutathione, and ascorbic acid, take effects in dependence of doses [23, 66, 67]. Additionally, it is reported that DOPAL plays a significant role in preventing α-synuclein aggregation, cytotoxicity and ROS production. NOD-like receptor family pyrin domain containing 3 (NLRP3) inflammasome in innate immune cells is persistently activated by accumulated misfolded α-synuclein and releases proinflammatory cytokines to trigger the death of DA neurons [38, 68, 69]. Therefore, above results demonstrate connections of PD in varied aspects: endotoxic catecholamines, oxidative stress neuroinflammation, and antioxidant effectors [70].

The study of Vagal application of DOPAL to stimulate PD-like autonomic dysfunction also revealed that mitochondrial swellings and hyperplasia deteriorated with the extension of administration time [62]. It can also be confirmed in Neu7 rat astrocyte cell line that DOPAL apparently reduced Neu7 viability, induced apoptosis, decreased mitochondrial performance and produced hydrogen peroxide and nitrite [71]. Its oxidative productions, like DOPAL quinones and DA-oxidated quinones, covalently modify mitochondrial protein, induce swelling and reduce respiratory activity, by the opening passage of 1-methyl-4-phenyl-1,2,3,6-tetra-hydropyridine [7, 26, 72]. Therefore, both DA and DOPAL-oxidated quinones activate apoptotic pathway. Meanwhile, the decrease of DOPAL-induced cell activity can be measured by extracellular lactate dehydrogenase concentration, an acknowledged index of necrosis [73, 74]. This toxic cascade was discovered in human PD neurons, but not in mice, at least partly due to species-specific differences in DA metabolism. Pathological phenotypes observed in human neurons can be recapitulated by the increased DA synthesis or the number of α-synuclein in the midbrain neurons of mice.

α-synuclein: It is discovered that α-synuclein has four different Mets, occurring in the form of Met sulfoxide (MetO) in the α-synuclein deposits due to high sensitivity to oxidation. In a study about a synthetic α-synuclein replaced all its Met by MetO moieties, MetO declines the affinity of α-synuclein towards SVs and inhibits the clustering and fusion of SVs [75]. Glycosylation of α-synuclein is proved to take the same effect [76].

Specifically, different forms of α-synuclein lead to different results. The excessive expression of wildtype (WT), A53T, or A30P α-synuclein results in reduction of spontaneous SV release [59]. Long-lasting potentiation and multidimensional ability are damaged by A53T α-synuclein [59]. Rab11 regulates synaptic trafficking and behaviour flaw [77]. Furthermore, α-synuclein can work with various SV proteins [78]. E46K α-synuclein mutants elevate lipid interactions and disrupt membrane selectivity in the simultaneous existence of VAMP2 which is necessary for α-synuclein-induced synaptic attenuation [78-80]. Synapsin III, a synaptic phosphoprotein that modulates DA release in collaboration with α-synuclein, exists in the Lewy bodies of PD patients and consists of the α-synuclein insoluble fibrils [81]. Its deficiency hinders α-synuclein polymerization, impairs striatal synapses and loses nigral cells [81].

Moreover, these changes of SV function precede neuron damages. But between wildtypes and mutants, there are no quantitative variance in total SVs or vesicles in the docked and reserve pool. Neurons exposed to α-synuclein fibrils, show reduced frequency and amplitudes of spontaneous Ca^2+^ transients, whereas synaptic activity remains stable [44]. Boosted tonic extracellular DA concentration and changed DA regulation of synaptic activity are found in mice with overexpression of human α-synuclein in the striatum, whereas there is inexistence of DA shortage [82].

As for alterations of α-synuclein, triggered by SV trafficking dysfunction and the accumulation of oxidized DA, increased α-synuclein is observed in mutant LRRK2 neurons [15]. Disrupted DA transmission led by genetic deletion of synaptic vesicle glycoprotein 2C (SV2C), damaged SVs and altered SVs pools induced by DOPAL, also results in the expression or oligomer formation of α-synuclein [40, 83].

## 4. Genetic Factors and Synaptic Vesicular Dysfunction in PD

**Figure 1.**
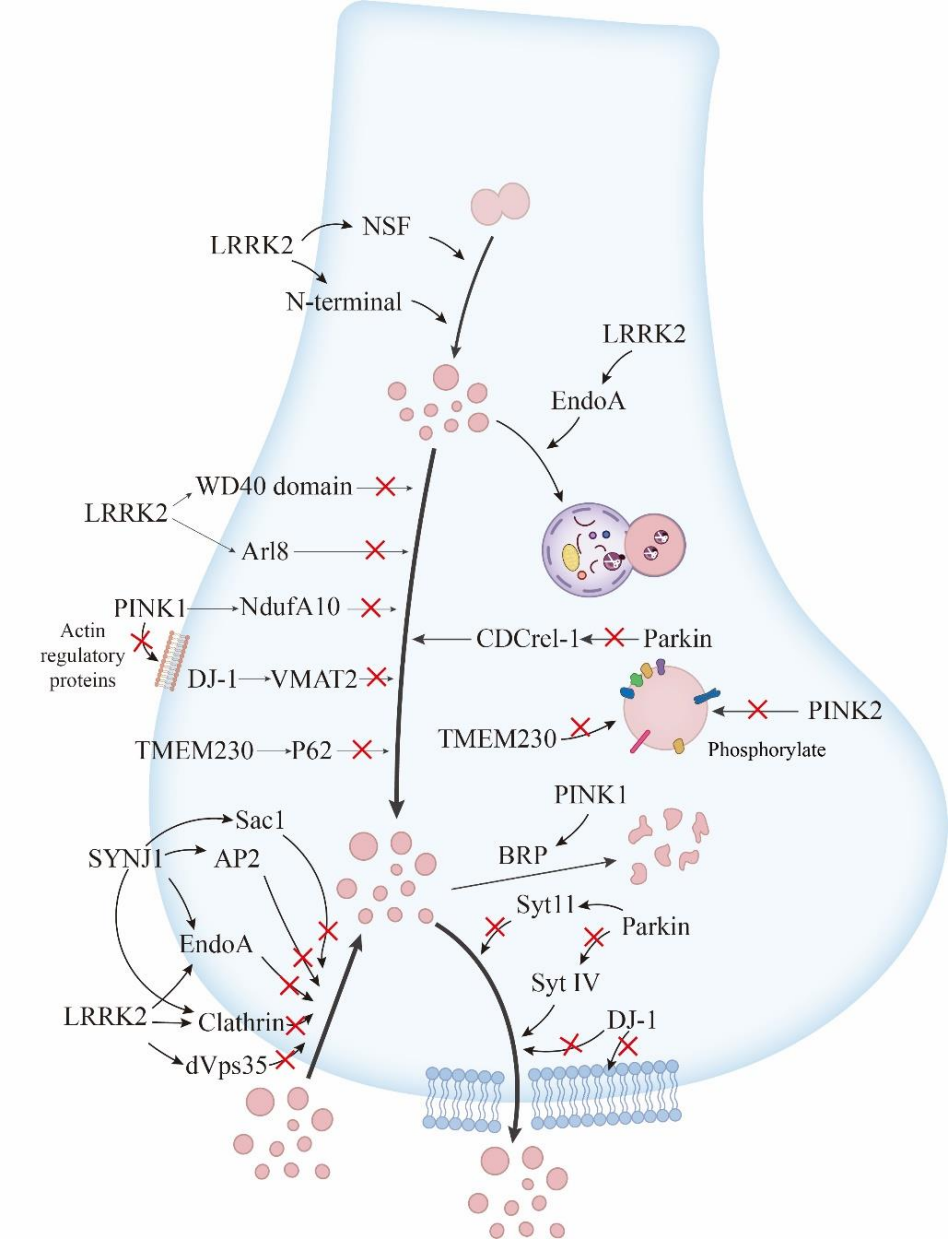
Different gene variants change the structure and quantity of related substances at the molecular level, thereby affecting the structure and function of synaptic vesicles.

Some accumulations of familial factors and missense mutants respectively serve to regulate synaptic function and dysfunction, such as α-synuclein, LRRK2, parkin, PTEN-induced kinase 1, and DJ-1. Gene mutations associated with altered SV function are summarized in Figure 1.

LRRK2: LRRK2, a multidomain protein including kinase, Guanosine triphosphatase, and altered protein interaction domains, plays an important role in PD family, such as mediating tubulation and vesicle sorting of lysosomes and inflammatory sensitization of THP-1 cells [54, 84]. It combines with neuron vesicles promoted by C-terminal WD40 domain and collaborates with vacuolar protein sorting 35 to modify synaptic vesicular endocytosis (SVE) to strike a balance of DA through the endosomal pathway [85, 86]. As a regulator of clathrin-mediated endocytosis, LRRK2 slows SV recycling in striatal neurons and impacts stockpile and mobilization within the recycling pool [87, 88]. Its function of dominating an Endophilin-A (EndoA) phosphorylation cycle in the endocytic process of synapses accounts for damages of functional endocytic regulation by its mutations [89, 90]. Its relation to the structural and functional regulation of synapses can be demonstrated by synaptic defects in dopaminergic neurons triggered by LRRK2 phosphorylation [15]. Resultant accumulation of α-synuclein produces ROS. Of note, even if the LRRK gene is not mutated, the wild LRRK protein may still activated by ROS to cause PD. The research of mice treated by H_2_O_2_ confirmed it and displayed the phosphorylation of Rab10 [91]. Dopaminergic neurodegenerative changes were found in the dependence of robust kinase and age in LRRK2 G2019S transgenic mice, including abnormally distributed vesicles and accumulated LRRK2 in mutated astrocytes [18, 92]. Kinase mutants triggered by LRRK2 hyperactivity destroy SV transmission and neurotransmitter release [93-95]. It can also lead to phosphorylation of pre-synaptic NSF protein to increasingly activate its adenosine triphosphatase (ATPase) and accelerate SNARE compound [96]. In the dependence of LRRK2, EndoA phosphoswitch is of great significance for macroautophagy at terminals of synapses [97]. LRRK2 R1441C modulates the SV proteome and phosphoproteome [98]. LRRK2 G2385R mutation partly influences SV transmission through altered protein interactions. N-terminal domain in the E193K mutant and C-terminal WD40 domain also take similar effects [99, 100]. Loss of Lrrk, a homologue of LRRK2, triggers accumulative lysosome-related organelle regulator, Arl8, resultantly impacting dense core vesicles at nerve endings [101].

Parkin: As an E3 ubiquitin ligase, Parkin mutates or conjugates with DA quinones into protein-bound quinone (quinoprotein) to trigger synaptic functional changes and early-onset autosomal recessive PD [25, 102, 103]. Similar symptoms with preclinical features of PD, such as decreased release of striatal evoked DA, abnormality in synaptic plasticity, and non-motor symptoms, have been revealed in a study about Parkin-KO mice [104]. By interacting with CDCrel-1, parkin modulates the release of SVs. CDCrel-1 is a protein in close correlation with synaptic vesicular dynamics. Mutant Parkin increases CDCrel-1 and hinders the release of neurotransmitter [105]. In addition, Parkin have been proved to impact the reuptake of SVs. It ubiquitinates synphilin-1, a protein which binds to α-synuclein to form inclusions, and then combines with synaptotagmin XI, a protein participating in the formation and docking of SVs. Loss of Parkin decreased the degradation of synaptotagmin XI, triggering accumulated synaptotagmin XI and damaged SVE. Moreover, in a study of injecting excessive synaptotagmin XI into mice, about 35% of DA neurons exhibited apoptosis [106-108].

PINK1: Inherited nonsense and missense mutations of PINK1 are proved to trigger the early-onset familial PD by Complex I activity decline and synaptic dysfunction [109]. For example, PINK1 deficiency inhibits dopaminergic neurons to develop normally, and triggers dyskinesia [110]. Although mice in shortage of PINK1 still develop dopaminergic neurons in normal quantity, the obviously declined release of DA demonstrates that PINK1 is involved in synaptic transmission. Transmission electron microscopy also confirms that PINK1 mutants trigger decreased and abnormal SVs at tetrad synapses in the visual system [111]. Therefore, it can be explained why Drosophila PINK1 mutant fails to maintain normal synaptic transmission during intense activity. In the brain, it possesses low-concentration postsynaptic density proteins (PSD95 and Shank), glutamate receptors (NR2B and mGluR5) and Bruchpilot (a presynaptic scaffolding protein of significance for all types of synaptic neurotransmission), and reduces abundance of other relevant proteins such as Rab5, Syntaxin and Wishful Thinking, except Synaptotagmin [111, 112]. In addition, activity and protein phosphorylation of PINK1 kinase but not its mitochondrial function, play an important role in remaining neuronal activity, which is confirmed by artificially caused neurodegeneration and declined protein phosphorylation in the brains of PINK1-defective monkeys without mitochondrial protein changes [113].

DJ-1: Mutation in DJ-1 is a known cause of early-onset autosomal recessive PD [114]. It is disclosed in localization studies that DJ-1 situates in the synaptic membrane, serving as a transducer for cellular redox homeostasis [115]. DJ-1 probably impacts synaptic functions, which is indirectly demonstrated by relatively reduced appetency for synaptic membrane via comparison between its pathogenic form and WT DJ-1. Moreover, DJ-1 controls sequestration and release of DA vesicles when depolarizing [116]. Its excessive expression diminishes oxidative reaction and enhances VMAT2 to protect dopaminergic neurons whereas its under-expression is counterproductive and damages SV endocytosis and reutilization at nerve terminals [19, 116]. Cholesterol levels may be a key pathway to modulate SVE by synaptic membrane fluidity. Jae Won Kyung et al proved that cholesterol deficiency damaged SVE in normal WT neurons while extra cholesterol restored SVE in DJ-1 knockout neurons [19]. In the PARK7-knockout mice, deficient DJ-1 had no obvious relation to the storage of catecholamine in SV, but it slowed the closing time of fusion pore to modulate duration and decay time of exocytosis [117].

Synaptojanin1 (SYNJ1): SYNJ1 serves as an enzyme giving full play to the phosphorylation and recycling of SVs, whose mutations can be found in a few families with early-onset atypical PD [118-121]. It is currently proved that inactive SYNJ1 probably damages SV recycle and triggers the onset of PD and other neurological diseases. For example, dystrophic dopaminergic axons and damaged synaptic clathrin uncoating resulting from Parkinson sac domain mutation in SYNJ1, and homeostatic imbalance of neuronal phosphoinositide caused by asparagine endopeptidase-mediated fission of SYNJ1, all lead to synaptic dysfunction [122, 123]. Li Zou et al found that neurodegeneration increased lysosomal permeability and promoted the leakage of Asparagine endopeptidase (AEP). As the substrate of AEP, SYNJ1 was cleaved at N599. SYNJ1 fragments remarkably reduced the miniature excitatory postsynaptic currents and inhibited the clustering of presynaptic vesicles in transgenic mice [123]. Other studies also showed that relevant signs of age-dependent dyskinesia, α-synuclein accumulation, damaged autophagy and dopaminergic terminal degeneration were manifested in male SYNJ1+/- mice [93]. Moreover, SYNJ1 can be phosphorylated by LRRK2 in vitro, leading to the impaired endophilin-synaptojanin1 interaction needed in SVE. Simultaneously phosphorylated SYNJ1 disrupts SVE and continuous exocytosis in midbrain neurons to change specific motor functions of 1-year-old male mice. Thus, it demonstrates that the cooperation between LRRK2 and SYNJ1 pathogenic pathways in impairing SV transmission in midbrain neurons probably plays a potential role in molecular mechanism of early-onset PD [93]. As for the genetic perspective, a new compound heterozygous mutation (p. Leu1406Phefs*42 and p. Lys1321Glu) in SYNJ1 gene invalid AP2 binding sites causes neuronal dysfunction of synapses and vesicular endocytic recycling [124]. Phosphorylation of SYNJ1 at S1163 enhances the SYNJ1 D5-phosphatase activity, alters the interaction between SYNJ1 and endophilin, and promotes the efficient endocytosis of the active cycling vesicle pool at the expense of reserve pool vesicle endocytosis [37,38].

Transmembrane protein 230 (TMEM230): TMEM230 was initially considered as a rare familial pathogenic gene, but afterwards it was found to be prone to impact PD risk [125-127]. It can encode two isoforms of TMEM230 proteins, with 183 amino acids (I) and 120 amino acids (II) [125]. TMEM230 is composed of two transmembrane segments whose N-terminal and C-terminal regions dissociate in the cytosol. The latter one occupies 95% of gross human protein isoforms [125]. To be specific, TMEM230 probably situates in neuronal vesicular structures, and its messenger ribonucleic acid is extremely active in many tissues, including the midbrain, neocortex, cerebellum, and spinal cord [125].

So far, it has been found four relevant TMEM230 mutations of PD [125]. Although its functions remain indistinct, TMEM230 is increasingly proved to play diverse roles in PD-onset cellular processes. First, TMEM230 co-localizes with proteins linking to SV transmission, including synaptophysin, VAMP2, VMAT2, and VPS35. Thus, relevant TMEM230 mutations in forms of knockout and low expression trigger dysfunction of SV transmission and trans-Golgi network secretion [128]. Second, TMEM230 co-immunostains with Rab5a, Rab11, and Rab8a, which take effects in endosomal recycle and transmission, vesicle secretion, and autophagy. Thus, phosphorylation of some of Rab family proteins by LRRK2 probably causes interaction between TMEM230 and LRRK2 [128]. Third, overexpression of isoform2 TMEM230 variants in SH-5Y5Y cells and mouse primary hippocampus neurons leads to comparably severe neurodegeneration and mitochondrial transmission impairment at the retrograde direction in axons [129]. In addition, TMEM230 mutation is also proved to improve concentration of a-synuclein [128] and trigger puncta or aggregation in SN4741 cells [130]. Conclusively, in consideration of unclear function of TMEM230 mutation in the PD-linked pathological process, exploring TMEM230 undoubtedly can provide a deep insight of the pathogenesis and ascertain new targets for intervention. The specific mechanisms of vesicle dysfunction induced by gene mutations in the above experiments are summarized in Table 1.

## 5. Environmental Factors and Synaptic Vesicular Dysfunction in PD

### 5.1 Impact of environmental factors on synaptic vesicular proteins

Studies have shown that besides genetic factors, environmental factors also have an impact on the onset and development of PD. A review reported that PD-relevant environmental factors can be divided into risk factors and protective factors. The former includes head injury, anxiety, and pesticides while the latter includes smoking, caffeine, and exercise. In terms of types, it is roughly composed of chemical substances, micro-organisms and behavioral habits [131-133]. Its pathological characterizations include increased concentration of iron in SN, accumulated extracellular neuromelanin (NM) and loss of DA neurons. Combining with superfluous Fe^3+^ and α- synuclein in succession, NM makes Ca^2+^ level out of balance or activates microglia to destroy DA neurons [134-137].

**Table 1:** Genes affecting DA homeostasis and the related mechanisms.

Gene	The influence on Synaptic vesicle	Outcome	Mechanism	Experimental animal	Refs
**LRRK2**	mediate tubulation and vesicle sorting from lysosomes	dynamically generate vesicles in damaged lysosomes.	the phosphorylation of Rab35 and Rab10 recruits JIP4 to lysosomes to promote the formation of LAMP1-negative tubules and release membranous content	Mice	[84]
**LRRK2**	influence SV endocytosis	N/A	dVps35 and dLRRK modulate synaptic endocytosis and SV regeneration via endocytic pathway mediated by Rab	Drosophila	[85]
**LRRK2**	Influence SV transmission	ectopic expression of the WD40 domain impacts transmission, distribution, and topology of the SV cycling pool.	WD40 domain interacts with SV-integral and -associated proteins	Mice	[86,99]
**LRRK2**	Influence SV endocytosis	too much and too little EndoA phosphorylation impairs synaptic endocytosis	EndoA phosphorylation	Drosophila	[89]
**LRRK2**	Influence SV endocytosis	LRRK2-G2019S mutation impairs synaptic endocytosis	Reduce Clathrin and endophilin	Mice, human	[90]
**LRRK2**	Influence SV endocytosis	change DA metabolism and dopaminergic neuronal toxicity	differential clathrin binding	Human	[15,87,88]
**LRRK2**	Impact the number of SVs and accumulation of CCV at DA terminals	DA neurons are killed in a kinase-dependent and cell-autonomous manner	N/A	Mice	[18]
**LRRK2**	change communication between astrocyte and neuron	The loss of DA neuron in a non-cell-autonomous contribution manner	change the morphology and distribution of MVBs, the morphology of secreted EVs, and over-accumulate LRRK2 in MVBs	Human	[92]
**LRRK2**	impact SV transmission	hyperactive kinase mutant impairs SV transmission	modulation of LRRK2 macro-molecular complex	Mice	[93,94]
**LRRK2**	impact SV fusion	aberrant phosphorylated NSF by mutant LRRK2 disrupts SV fusion	phosphorylates pre-synaptic NSF	Mice	[95,96]
**LRRK2**	accelerates autophagy at the synapse	deregulation of Endophilin A phosphorylation promotes neurodegeneration.	Endophilin A phosphorylation recruits Atg3	Drosophila	[97]
**LRRK2**	SV proteome and phosphoproteome	Less generation of SV and DA neuron loss	phosphorylate SV proteins	Drosophila	[98]
**LRRK2**	promotes fusion	N/A	N-terminal domain mutation modifies the LRRK2-protein interactome and reduces its combination with SV	Mice	[100]
**LRRK2**	Influence SV transmission	dense core vesicles at the most distal boutons of the neuron terminals	Arl8 accumulation	Drosophila	[101]
**Parkin**	Influence exocytosis	Promote neurotransmitter release	Parkin overexpression degrades Syt IV protein, but not Syt I protein	N/A	[104]
**Parkin**	Influence SV transmission	N/A	CDCrel-1 degradation	mice	[105]
**Parkin**	Influence exocytosis	reduce vesicle replenishment and DA release	Parkin dysfunction triggers accumulative Syt 11	mice	[106]
**PINK1**	Influence SV transmission	PINK1 mutant impair SV transmission	NdufA10 ubiquinone uncoupling	Drosophila Mice	[109]
**PINK1**	Influence the number, shape, and transmission of SV	longer sleep during the day, shorter lifespan and decreased climbing ability	reduce BRP and other relative proteins	Drosophila	[111]
**PINK1**	Influence postsynaptic plasticity	neurodegeneration and cognitive decline	reduce SV-relative proteins, regulate dendritic spine morphology by actin regulatory proteins and regulate neuronal survival by decreased Akt activation	Mice	[112]
**PINK1**	N/A	severe neuronal loss	reduce protein phosphorylation	Mice Monkey	[113]
**DJ-1**	sequestration and transmission of DA in SV	early-onset familial PD	DJ-1 mutant reduces VMAT2	Mice	[116]
**DJ-1**	Influence SV endocytosis	decreased DJ-1 damages SV endocytosis and reavailability	regulate membrane cholesterol to regulate synaptic membrane fluidity	Mice	[19]
**DJ-1**	exocytosis	prolongs exocytosis	Increase aggregation of α-synuclein	Mice	[117]
**SYNJ1**	Influence SV recycling	recycling dysfunction	phosphoinositide metabolism and combination with amphiphysin	N/A	[118]
**SYNJ1**	Influence SV endocytosis	damage SV function and trigger early-onset Parkinsonism	damage phosphorylation of Sac1 domain substrates	Human	[119, 121]
**SYNJ1**	Influence SV endocytosis	endocytic impairment and accumulation of clathrin-coated intermediates	damage Clathrin Uncoating	Mice	[122]
**SYNJ1**	Influence SV endocytosis	damage phosphoinositide homeostasis and trigger synaptic dysfunction	AEP cleavages SYNJ1	Mice	[123]
**SYNJ1**	Influence SV endocytosis	SV transmission damage in terminals of MB neuronal but not cortical neurons	Phosphorylated by LRRK2	Mice	[93]
**SYNJ1**	Influence SV endocytosis	juvenile atypical PD	damage the AP2 binding sites	Human	[124]
**TMEM230**	encode a transmembrane protein of secretory/recycling vesicles	Damage SV transmission	N/A	N/A	[125]
**TMEM230**	Influence transmission of secretory vesicle and retromer	retromer mislocalization, cargo transmission and damaged autophagy	inhibits extracellular secretion of p62 and immature lysosomal hydrolases in Golgi-derived vesicles, mediated by loss of the small GTPase Rab8a	Human	[128]
**TMEM230**	N/A	neurodegeneration and damaged axonal mitochondrial transmission	N/A	Mice	[129]

Abbreviations: N/A: not available; SV: synaptic vesicle; PD: Parkinson’s disease; JIP4: JNK-Associated Leucine-Zipper Protein 4; LAMP1: lysosome-associated membrane protein 1; dVps35: Drosophila Vps35; dLRRK: Drosophila LRRK2; EndoA: Endophilin-A; CCV: clathrin-coated vesicle; MVBs: multivesicular bodies; NSF: N-ethylmaleimide sensitive fusion; Syt IV: Synaptotagmin-IV; Syt I: Synaptotagmin-I; NLRP3: NOD-like receptor family pyrin domain containing 3; Atg3: autophagy-related gene 3; Dex: dexmedetomidine; AMPK: AMP-activated protein kinase; NdufA10: NADH: ubiquinone oxidoreductase subunit A10; BRP: Bruchpilot; VMAT2: Vesicular Monoamine Transporter-2; AEP: asparagine endopeptidase; MB: midbrain; GTPase: Guanosine triphosphatase; SYNJ1: Synaptojanin1; TMEM230: transmembrane protein 230; DJ-1:; PINK1: PTEN-induced putative kinase1; LRRK2: Leucine-rich repeat kinase 2.

A recent study whose research objects were exposed to methamphetamine in a long time, reveals a remarkable increase of DOPAL and decrease of DA receptors [138]. Moreover, drug addicts were sensitive to a neurotoxin in herbicides, 1-methyl-4-phenyl-1,2,3,6-tetrahydropyridine [139]. Paraquat with similar characteristics was 3 times more in risk to develop PD [140]. Some pesticides induced PD through damaging aldehyde dehydrogenase and increasing α-synuclein [141, 142]. Another pesticide, rotenone, raised reactive oxygen and decreased ATP by inhibition of mitochondrial Complex 1 to increase 2.5-fold risk of PD onset, even used in preparing PD models [143, 144]. Exposing to rotenone, the T-bar and SV attaching platform of Drosophila are smaller. Its SVs are shaped abnormally and become more transparent and easier to broke [145]. Pesticide maneb was proved to modulate synaptic vesicular proteins in mice, such as elevated level of dopamine transporter (DAT) and D (1 A) dopamine receptor and reduced TH [146]. A study of mice administered by glyphosate displayed that DAT, striatal acetylcholinesterase and antioxidant reaction were all inhibited, and the release of DA by SV exocytosis was promoted [147].

Some experts considered that α-synuclein related pathology derived from the nose and gastrointestinal tract. Proliferating significantly in PD patients, intestinal flora has been considered to impact the development of PD through gut permeability and systemic inflammatory responses [142, 148, 149]. The coronavirus, which had respiratory and gastrointestinal symptoms, was suspected to transmit to the brain through the olfactory bulb and vagus nerve. SARS-CoV-2 Spike S1 protein receptor binding domain combined with heparin and heparin binding proteins which promoted the aggregation of α-synuclein and the onset of PD [150-153].

Moreover, in the co-existence of divalent metal ions and DOPAL, α-synuclein aggregation increased significantly while DA or other DA metabolites did not play a similar role [154]. Copper ions, as catalysts, destroyed hydrogen atoms and removed electrons from DOPAL's aldehyde admixtures, taking major effects in the formation of α-synuclein aggregation. Significantly, Fe^2+^ and Cu^2+^ both increased α-synuclein oligomerization but Fe^3+^ and Cu^+^ did not take the same effect. Thus, it was considered that the promotion of α-synuclein oligomerization depended on the divalency of metal ion rather than their redox states [155-157]. Under the condition of low Cu^2+^ concentration, the chain of stable trimers was lengthened by binding monomers laterally, but at high concentration, the broken stability resulted in rearranging conformation and reforming annular oligomers [158].

Interestingly, smoking was proved to be negatively correlated with the risk of PD. The researchers did an experiment and found that in comparison with control groups, striatal DAT binding and the volume of smokers’ gray matter both obviously decreased, suggesting that nicotine probably enhanced the effect of DA and damaged striatal DAT [159]. Another controlled experiment also reached a similar conclusion of an inversed association between long-term coffee consumption and striatal volume in current coffee consumers [160].

### 5.2 Interaction between environmental factors and genetic susceptibility in PD

Both genetic and environmental factors have been found to be closely related to the onset of PD. However, genetic factors only account for a small percentage, and environmental factors are not exposed to all patients. Therefore, a single factor is not enough to cause the onset of PD [131, 161]. Some researchers have confirmed that changes in genetic factors lead to increased sensitivity of individuals to environmental factors [162, 163]. M J Casarejo et al exposed the neurons from Parkin-knockout mice to rotenone and reached a conclusion that gene knockout made the neurons more susceptible to low doses of rotenone [163]. In other studies of PD-relevant gene knockin mice, experimental groups exhibited normal consequences in motor activity and number and morphology of DA neurons under normal conditions. But on condition that they were exposed to reserpine or rotenone, the experimental groups were more likely to manifest motor impairment, DA uptake damage, ATP shortage and apoptosis [164, 165].

In addition, mitochondrial dysfunction has been found in both genetic and environmental factors induced PD patients. Therefore, it is proposed as the co-pathogenic pathway and the key to the interaction between the two mechanisms [166]. Enormous studies revealed that the neurons from PD patients with gene defects exhibited abnormalities in number, proportion, morphology, structure and mitophagy of mitochondria [167-170]. Mitochondrial dysfunction makes neurons more sensitive to oxidation and metal ions [171, 172]. To be specific, the neuroprogenitors from PD patients with PARK2 gene dysfunction displayed greater sensitivity to the toxicity of Cu and decreased minimal effect concentration to 10 μm. Exposure to Cu aggravated the breakup of mitochondria [162].

### 5.3 Role of synaptic vesicular dysfunction in mediating environmental risk factors in PD

The density and distribution of presynaptic vesicles, which are represented by the level of synaptophysin (SYP), indirectly demonstrate the quantity and distribution of synapses [173]. A reduced concentration of SYP damages the transport of SV and the transmission, processing and storage of neural signals [174]. Postsynaptic density protein-95 (PSD-95) takes effects to support and anchor postsynaptic receptors [175]. Researchers found that mice exposed to Cu exhibited decreased level of SYP, PSD-95, and other transmitters such as DA, 5-HT and GABA, accounting for its negative effects in the pre and post synaptic regulation and release of transmitters. Moreover, Cu inhibits synaptic conduction by decreasing post-synaptic dense substances or inactivating synapses [174].

In addition, Cu-induced oxidative stress was also proposed to involve in synaptic dysfunction. Brain-derived neurotrophic factor (BDNF) is an important target of cAMP-responsive element binding protein (CREB), taking effect in regulating synaptic plasticity and the function of CREB on the condition of mediating CREB transcription factor. The transcription factor promotes the expression of BDNF, and the latter combines with receptor TrkB to activate CREB [176-178]. CREB was proved to damage synaptic function by its abnormal phosphorylation and transcription machinery [179, 180]. Some relevant studies considered Cu-induced oxidative stress inactivated the phosphorylation of CREB and decreased the level of the downstream proteins BDNF and TrkB, leading to the damage of synaptic plasticity [174, 181].

## 6. Converging Mechanism: Disrupted Vesicular Dynamics in PD Pathogenesis

### 6.1 Interaction between altered synaptic vesicular proteins and dopamine dysregulation

Excessive DA in the cytosol generates DA quinone and superoxide by autooxidation or enzymatic oxidative reaction [182]. If not oxidated, it is sensitive to be catabolized by enzymes to develop DOPAL and H2O2 [183]. Its metabolites in two pathways have been proved to damage neuronal structure and mitochondrial functions [7, 184]. To avoid DA deposits, VMAT2 sequesters resynthesized or reingested DA into SVs to stabilize DA homeostasis. Thus, VMAT2 shortage in PD triggers DA dysregulation. A set of autopsy data from PD patients showed deficient VMAT2, decreased vesicular uptake of DA and increased DA metabolites [185, 186]. Bucher et al designed a new mice model with VMAT2 deficiency by viral-mediated small-hairpin RNA interference. It reached conclusions of a 44% decrease of VMAT2, a 50.4% loss of DA, a 64.7% increase of DA turnover, and a 27.4% increase of DA oxidation [187]. These results indicate that the alteration of synaptic vesicular proteins can inhibit the transport of DA and cause its abnormal metabolism.

Dunn et al proposed a SV2C-knockdown mice model by guiding small-hairpin RNA into Neuro-2a cells, also exhibiting a 32% reduction of DA release. Serving to modifying L-DOPA and nicotine, SV2 is considered to participate in regulating synaptic function and mediate DA homeostasis. Moreover, to explore whether long-term DA dysfunction leads to alteration of SV proteins, researchers assessed the amount of SV proteins in animals with a low DA level. In these studies, expression of VMAT2 was greatly inhibited, which triggered deficient vesicular storage of DA. It was found that there were no significant changes in SV2C and various endocytic proteins [83, 188]. In addition, SV2C takes an effect in modulating the PD-relevant environmental factor nicotine to recover neurodegeneration, becoming a new target of PD treatment [189].

### 6.2 Impact of disrupted vesicular dynamics on neurodegenerative changes in PD

PD is featured with degeneration and loss of DA neurons and resultant symptoms such as tremor and rigidity [190, 191]. It has been confirmed that synaptic dysfunction occurs before impairment of DA neurons [192]. Thus, damaged vesicular dynamics is considered as a justification for neurodegenerative changes in PD.

In the VMAT2-deficient mice model proposed by Bucher, DA transmission into vesicles is reduced and DA neurons decreased by 40.8%. At 20 weeks after the model was established, the mice showed significant differences in behavioral tests compared to the normal. It is in part because DA neurons are susceptible to DA deposits in the cytosol. The deposited DA is prone to produce oxidation products that destroy neurons. Thus, researchers assessed the degree of oxidative damage to proteins. It was found a 26.7% increase of 4-hydroxynonenal and a 27.6% increase of 3-nitrotyrosine, both of which were common indicators of oxidative stress and suggested that destroyed synaptic vesicular transport triggered neuronal degeneration [190, 193]

SVE dysfunction also participates in degeneration of presynaptic nerve terminals. SV is regenerated through SVE and ingests DA by a vATPase-created proton gradient on SV membrane surface. Under normal circumstances, vATPase is restrained by the clathrin coat and is activated by the uncoating of auxilin. The uncoated clathrin-coated vesicles (CCVs) transform into SVs, loading with neurotransmitters and completing exocytosis. Otherwise, impaired SVE decreased DA packing into vesicles, also resulting in the deposition of DA in cytoplasm and neurodegeneration [194-197]. In addition, α-synuclein may impacts the formation and fission of CCVs [190, 198]. lack of α-synuclein decreased the amounts of vesicles keeping away from the active presynaptic region [199]. The amounts of circulated vesicles are not kept in timely maintenance and replenishment [200]. But acute stimulation of neurons by injection of α-synuclein remarkably inhibits SVE, triggering accumulated CCVs, deposited DA and final neurodegeneration [201].

### 6.3 Putative central mechanism linking synaptic vesicular dysfunction and PD pathogenesis

Due to impaired peripheral or central nervous system including the vagus nerve, the sympathetic nerve, or the olfactory bulb, symptoms of neurodegeneration occur during the early period [202]. SV transmission dysfunction triggers the deposition and oxidation of DA, leading to increased aggregations of α-synuclein which are characteristic performance of PD neurodegeneration [203]. Abnormal α-synuclein spreads through the brain in two ways, from the glossopharyngeal nerve and the vagus nerve to the central nerve or directly between central neurons in a prion-like way [202, 204]. Finally, it inhibits the activity of various brain regions in PD patients, such as amygdala, hippocampus, insula and medial frontal gyrus [205-208].

SV dysfunction-induced α-synuclein remarkably activates microglia, resulting in excessive expression of HLA-DR, a receptor on the surface of DA neurons. HAL provides digested antigenic peptides to facilitate the recognition of CD4+ T lymphocytes which infiltrate the brain through the relatively permeable blood-brain barrier in PD patients. Simultaneously, inflammatory molecules secreted by microglia also promote the presentation of antigens, proliferation of immune cells, and final neural apoptosis. Therefore, synaptic dysfunction leads to the onset of PD by overexpression of inflammatory molecules [209].

After accepting signals of impaired neurons, astrocytes secret neural protective factors to inhibit neurodegeneration [210]. Therefore, DA neurons in the brain regions with less astrocytes are prone to degenerate. Abnormal α-synuclein released from damaged neurons are absorbed and degraded by astrocytes. The amount of α-synuclein in the astrocytes is in direct proportion to the severity of DA neuron loss [211-213]. Thus, α-synuclein centralizes in sensitive areas of PD, such as amygdala, thalamus and striatum [214]. α-synuclein activates microglia to secret inflammatory factors and then induce A1 astrocytes. A1 astrocytes have no ability of promoting growth, recovery or normal synaptic function but triggering neuroapoptosis [215]. Its modulation for stability of BBB is destroyed by α-synuclein. it secrets proinflammatory cytokines and induces microglia to generate inflammatory reactions [216]. The two-way stimulation of microglia and astrocyte leads to neurodegeneration [216, 217].

## 7. Therapeutic Implications and Future Directions

### 7.1 Potential therapeutic targets based on synaptic vesicular dysfunction

DA replacement is often used to treat PD patients. but it only takes a transient effect and is not available to recover DA neurons. Thus, exploring a new method is urgent.

LRRK2 has been shown to phosphorylate Ras-associated binding protein (Rab) by activation of LRRK2 kinase [218, 219]. To be specific, SV release is modulated by Rab3a and Rab27b, recycling by Rab4 and Rab7, retrieval by Rab5, anterograde axonal transport by Rab11 and Rab22, retrograde axonal trafficking by Rab5 and Rab7, degradation and turnover by Rab26 and Rab33. [218, 220-225]. Mutant LRRK2 greatly affects vesicle dynamics. Taking effects in the adenine region of ATP and the ATP-binding site, LRRK2 inhibitors dephosphorylate Ser910 and Ser935 residues and decrease LRRK activity [226, 227]. Many inhibitors have been developed and put into clinical trials with positive responses [228]. Thus, it has a high research value and prospect [229].

If synaptic transmission encounters the difficulty of vesicle shortage, reduced neurotransmitter release would destroy the fidelity of neuronal signalling [230]. Ca^2+^-dependent recovery (CDR) was considered as a solution by expediting vesicle replenishment [231]. Among them, synaptotagmin 3 (SYT3) possessing high affinity with Ca^2+^ was a key target [232]. A recent study about the immunolabeling experiment of mouse brain stem showed that SYT3 abounded in the presynaptic active region. A remarkable depression was found in the synapses of SYT3-knockout mice by stimulating axons with 100-stimuli trains and its speed of recovery was twice as slow as the normal. By injecting an viral vector with SYT3 gene, synapses was faster recovered and exhibited an obvious improvement in depression, which demonstrated that SYT3 played a key role in sustaining neurotransmitter release [230]. In addition, only binding with Ca^2+^ can SYT3 promptly transform vesicles into tightly docked or active states [233].

Moreover, auxilin promotes SV regeneration by uncoating of CCVs, a common target in various endocytic pathways. Auxilin-knockout mice was found to possess a greater proportion of clathrin cages. Of the 49 proteins with significant changes, reduced 39 proteins were all SV transmembrane proteins. It revealed that imbalance in CCV/SV ratio destroyed SV sorting. Auxilin shortage also triggers excessive synaptic autophagy and incorrect transmission of DAT which hinders protein trafficking in axonal membrane whirls, thereby leading to damaged DA homeostasis [13, 188, 234]. In the field of cancer, Ultrasound Microbubble Treatment is used to promote clathrin-mediated endocytosis, facilitating the delivery of targeted drugs. It may be a reference for PD treatment [235].

### 7.2 Novel treatment strategies focusing on restoring dopamine homeostasis

As is well-known, Levodopa is the raw material that produces DA. L-DOPA is usually administered less but more frequently to disperse its dose and stabilize its concentration level [236]. Dopamine agonists can overcome the disadvantage of a short half-life on L-DOPA, hindering from dramatical fluctuation of DA homeostasis [237]. Besides, COMT and MAO-B inhibitors also take an effective role in restoring DA homeostasis. They retard the degradation of L-DOPA and DA, stabilizing the concentration level of endogenous DA [238]. Of note, the COMT inhibitor is only used in combination with L-DOPA [239].

Studies have shown that DOPAL induced protein modification is dependent on DOPAL oxidation [65]. Antioxidants have been used to inhibit the oxidation of DOPAL to reduce the production of DOPAL metabolites. N-acetylcysteine (NAC) reduced the formation of quinone, impairing or blocking modifications of proteins and quinonization of partial proteins in a low molecular weight triggered by DOPAL [240]. By means of near infrared fluorescence spectroscopy, researchers found that in the experiment of MO3.13 and PC12 cells, antioxidant NAC suppressed all the above-mentioned alterations of proteins in their forms and functions [23]. Moreover, the quinonization and oligomerization were proved to be dominated by lysine and could be intercepted by treatment with citrate glycosides [40, 67].

Inhibition of α-synuclein aggregation and destruction of aggregates are also major treatment strategies. In condition of co-incubating the α-synuclein and the metabolite of Hydroxytyrosol, 4-hydroxy-3-methoxyphenylethanol, the fluorescence signal was weakened and the formation of fiber was reduced by Thioflavin T fluorescence method, which both demonstrated the decreased concentration level of α-synuclein [38]. In addition, nanoparticle is one of the major research directions at present. Recent studies have revealed that in the microglia, Cu_2-x_ selenium anti-Transient Receptor Potential Vanilloid type 1 (TRPV1) nanoparticles open TRPV1 channels after irradiated by near-infrared laser twice, leading to Ca^2+^ inflow. The phosphorylation of AMPK protein was resultantly activated and triggered intensive autophagy. Compared with the control group, α-synuclein remarkably and rapidly degraded [241, 242].

### 7.3 Future research directions and areas of investigation

Current major treatments for PD include antioxidants, mitochondrial therapy, targeted nucleoprotein therapy, and inhibition of neuroinflammation. Carbidopa and dopamine agonists are often used in place of DA but can cause many side effects, such as dyskinesias [243-246]. Moreover, current treatments are primarily aimed at treating symptoms rather than underlying neuro-degeneration [247]. Thus, we need to focus more on finding new research directions.

The blood-brain barrier is a semi-permeable membrane composed of endothelial cells and surrounding brain microcirculation. Its high selectivity physically inhibits access to cells and molecules. Therefore, the effect of traditional drug therapy is not ideal. However, the use of nanomaterials to carry and transport drugs may be a breakthrough in the puzzle [248]. The application of nanomaterials will help to reduce the frequency of administration and toxicity. In the recent clinical trials using nanomaterials, the majority ended in failure due to defects in its inherent properties. This suggested that the design for its specific delivery and toxicity characteristics still needed to be completed [242].

Natural resources are speculated as possible sources of new drugs to overcome the adverse reactions triggered by traditional medicine [246]. Some herbal extracts have been found to take an effect of suppressing the development of PD [246, 249]. For example, a recent study demonstrated that eupalinolide B passively targeted Ubiquitin-specific protease 7 and led to degradation of Keap1. The resultantly activated Nrf2 enhanced the expression of antioxidant response element-dependent gene and restrained neuroinflammation [250]. But the extraction and purification of Chinese herbal ingredients are not precise, so it will be the focus of future research.

In recent years, researchers have focused on regenerative medicine therapies, encompassing cell replacement and tissue engineering. It depends on specific construction of dopaminergic neurons which are differentiated from autologous stem cells. Based on the methodology of induced pluripotent stem cells, it can achieve the effect of functional recovery and decrease immunosuppression simultaneously [247]. However, the low success rate of transplanted midbrain dopamine neurons (mDANs) bothers researchers and blocks the development of novel treatment strategy [251]. A recent study showed that surgical operations lead to dramatical responses including acute neuroinflammation, severe infiltration of peripheral immune cells and even brain cell death. Moreover, co-transplantation of Treg cells was found to raise the success rate of transplanted mDANs and generate a larger number of TH+ transplanted neurons [252].

## 8. Discussion

PD is a common neurodegenerative disease with functional disruptions of motor and non-motor. It is associated with a progressive decline of dopaminergic neurons in SN and VTA. This decline is considered to occur through retrograde degeneration of the axonal projections in the striatum. Overall, this progressive degeneration results in loss of nigral nerve terminals cells and their projections, marked reduction in DA release and synaptic dysfunction in the striatum, manifesting as motor dysfunction characterized by bradykinesia, rigidity and resting tremor. The pathogenesis of PD is unclear but is thought to involve a complex process of aging, environmental factors and genetic susceptibility. Up to now, some neurotoxic results have been imputed to DA which plays a dangerous role under the influence of the synergy between the catechol and aldehyde moieties, exponentially intensifying the negative effects of DA regulation. This would perfectly conform to the multiple-hit scenario.
